# Dynamic Motion and Communication in the Streptococcal C1 Phage Lysin, PlyC

**DOI:** 10.1371/journal.pone.0140219

**Published:** 2015-10-15

**Authors:** Blake T. Riley, Sebastian S. Broendum, Cyril F. Reboul, Nathan P. Cowieson, Mauricio G. S. Costa, Itamar Kass, Colin Jackson, David Perahia, Ashley M. Buckle, Sheena McGowan

**Affiliations:** 1 Biomedicine Discovery Institute and Department of Biochemistry and Molecular Biology, Monash University, Clayton, Australia; 2 Australian Research Council Centre of Excellence in Advanced Molecular Imaging, Monash University, Clayton, Australia; 3 Australian Synchrotron, Clayton, Australia; 4 Programa de Computação Científica, Fundação Oswaldo Cruz, Rio de Janeiro, Brazil; 5 Victorian Life Sciences Computation Initiative Life Sciences Computation Centre, Monash University, Clayton, Australia; 6 Research School of Chemistry, Australian National University, Canberra, Australia; 7 Laboratoire de Biologie et de Pharmacologie Appliquée, Ecole Normale Supérieure de Cachan, Centre National de la Recherche Scientifique, Cachan, France; 8 Biomedicine Discovery Institute and Department of Microbiology, Monash University, Clayton, Australia; George Washington University, UNITED STATES

## Abstract

The growing problem of antibiotic resistance underlies the critical need to develop new treatments to prevent and control resistant bacterial infection. Exogenous application of bacteriophage lysins results in rapid and specific destruction of Gram-positive bacteria and therefore lysins represent novel antibacterial agents. The PlyC phage lysin is the most potent lysin characterized to date and can rapidly lyse Group A, C and E streptococci. Previously, we have determined the X-ray crystal structure of PlyC, revealing a complicated and unique arrangement of nine proteins. The scaffold features a multimeric cell-wall docking assembly bound to two catalytic domains that communicate and work synergistically. However, the crystal structure appeared to be auto-inhibited and raised important questions as to the mechanism underlying its extreme potency. Here we use small angle X-ray scattering (SAXS) and reveal that the conformational ensemble of PlyC in solution is different to that in the crystal structure. We also investigated the flexibility of the enzyme using both normal mode (NM) analysis and molecular dynamics (MD) simulations. Consistent with our SAXS data, MD simulations show rotational dynamics of both catalytic domains, and implicate inter-domain communication in achieving a substrate-ready conformation required for enzyme function. Our studies therefore provide insights into how the domains in the PlyC holoenzyme may act together to achieve its extraordinary potency.

## Introduction

Resistance to our current antibiotics is reaching crisis levels and is considered a serious threat by world health officials [1–3]. There is a growing need for investment and research into antibacterial agents with new modes of action. Bacteriophages are viruses that specifically infect bacteria, resulting in cell wall lysis and destruction of the host bacterium [4]. As part of the phage lifecycle, lysin proteins are produced to hydrolyze the peptidoglycan cell wall resulting in cell rupture and concomitant virus release through loss of osmotic integrity. Purified lysins as proteinaceous antimicrobials represent an excellent way to harness millions of years of evolution of bacteriophage. Exogenous application of lysins results in rapid and specific destruction of Gram-positive bacteria, making the purified lysins functional "inside-out" enzymes [5,6].

The streptococcal C1 phage lysin, PlyC, is the most potent lysin described to date, with a specific activity ~100 fold that of the next most catalytic lysin [7]. Previous research spanning over 50 years has shown that PlyC can rapidly lyse cultures of groups A, C, and E streptococci in addition to *Streptococcus uberis* and *S*. *equi* [7,8]. Whereas microgram or milligram quantities of most phage lysins can effect a multiple-log drop of target bacteria in minutes, PlyC requires only nanogram quantities to reduce mice mucosally colonised with 10^7^ *S*. *pyogenes* by >6 logs only seconds after enzyme addition [7].

**Fig 1:**
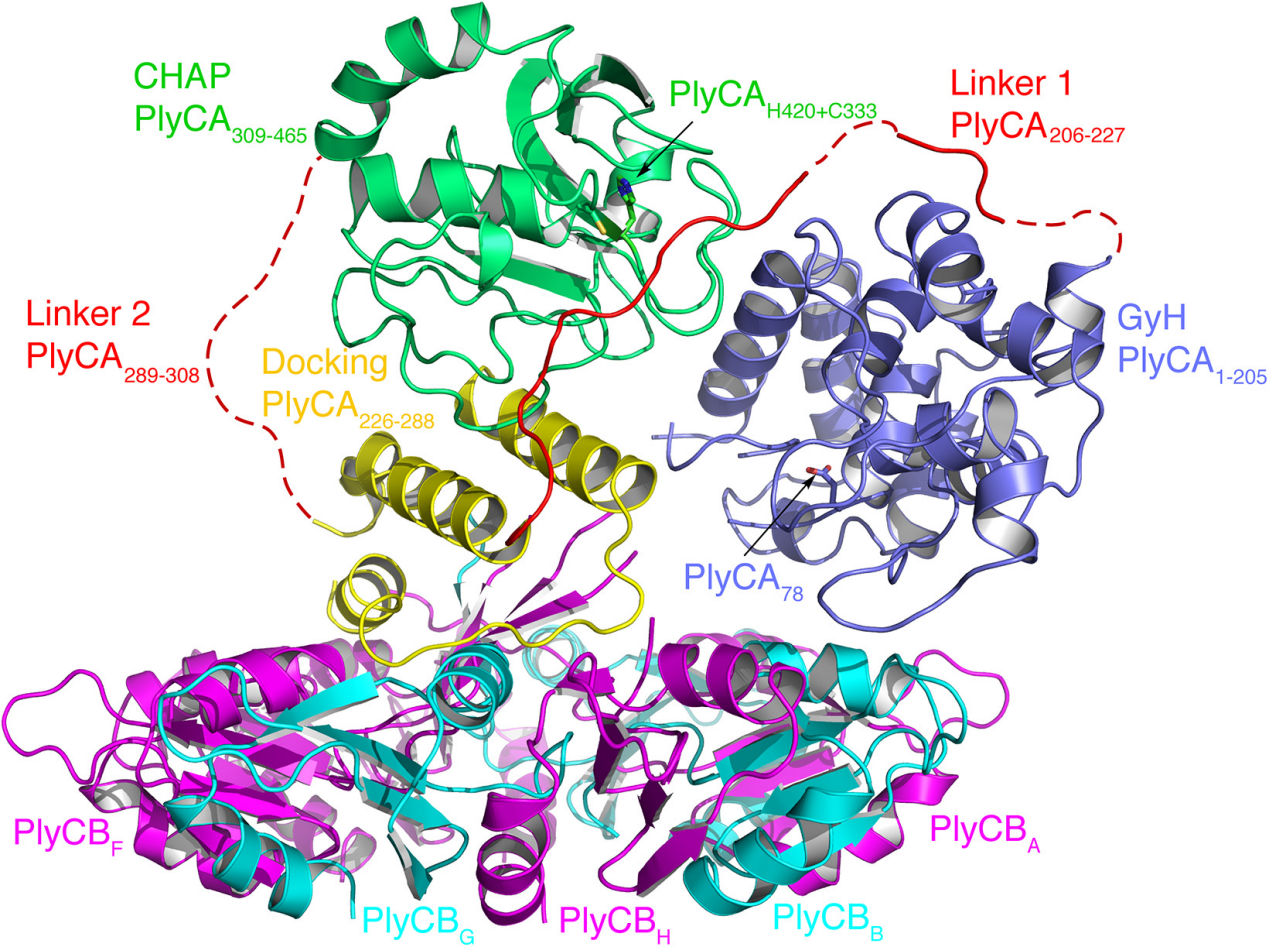
The molecular architecture of PlyC. The different domains of the PlyCA molecule are coloured as indicated. The N-terminal GyH domain (residues 1–205) is shown in light blue, the disordered linker 1 (residues 206–227) in red, the helical structure (residues 226–288) that docks PlyCA to PlyCB in yellow, the second disordered linker 2 (residues 289–308) as a dashed red line, and the CHAP domain (residues 309–465) in green. PlyCB monomers are colored in magenta and cyan alternatively and labeled A–H. Regions of disordered/absent density are depicted by dashed lines. Adapted from (McGowan et al. 2012).

PlyC is unique amongst the Gram-positive lysins in that it is a multimeric lysin composed of two distinct gene products, PlyCA and PlyCB [9]. The X-ray crystal structure of the complete PlyC holoenzyme, recently determined in our laboratory, revealed that the functional biological assembly consists of a single PlyCA protein bound to a ring-shaped assembly of eight PlyCB molecules (Fig 1) [10]. The symmetry of the planar octamer is broken by four copies of the PlyCB monomer that each contribute their first eight N-terminal residues to form a central β-sheet that extends up from the centre of the PlyCB assembly. Docked onto this surface is a single copy of PlyCA. The PlyCA protein is modular, consisting of two separate enzymatically active catalytic domains separated by a central docking domain. At the N-terminus of the PlyCA protein is the **G**l**y**cosyl **H**ydrolase (GyH) domain that is responsible for cleaving the sugar bonds of peptidoglycan through its glycosidase activity. In the middle of the PlyCA protein is a helical docking domain. This unique docking platform interacts with the PlyCB central β-sheet, forming a tight hydrophobic interface that acts as the protein-protein interaction for the assembly. At the C-terminus of PlyCA is the **C**ysteine, **H**istidine-dependent **A**midohydrolase/**P**eptidase (CHAP) domain, capable of cleaving both the sugar bonds as well as peptide bonds present in peptidoglycan. Connecting the two catalytic domains, GyH and CHAP, to the central docking domain are two linker regions (Fig 1).

Intriguingly, in the crystal structure of PlyC the active sites of both catalytic domains are occluded; catalytic residues PlyCA_H420_ and PlyCA_C333_ of the CHAP domain are occluded by linker region 1, which connects the CHAP and docking domains; the orientation of the GyH domain buries its active site at the interface with the PlyCB ring (Fig 1). Taken together, these observations imply that an inactive, autoinhibited conformation of the enzyme was crystallized [10]. Whilst X-ray crystallography is powerful, the structures determined using this technique are essentially static snapshots, containing limited information on how molecules move. It is firmly established that protein dynamics and flexibility play important roles in governing biological function (eg see [11] for a review). To complement and extend crystallographic analyses, molecular dynamics (MD) simulation is now an established approach for characterizing the conformational properties of a system [12–16]. In this study we use MD simulations combined with small angle X-ray scattering (SAXS) to investigate how PlyC achieves a functionally-competent conformation, providing insights into the remarkable catalytic efficiency of this complex machine.

## Materials and Methods

### Production of PlyC

The PlyC protein was produced as described previously [7].

### Small Angle X-ray Scattering (SAXS)

SAXS measurements were made at the SAXS-WAXS beamline of the Australian Synchrotron, Melbourne, Australia. For each SAXS measurement, 10x1 second exposures were measured and averaged together after verifying that there was no evidence of radiation damage (systematic change in the shape of the scattering curves as a function of exposure time). During collection the sample was flowed through a 1.5 mm quartz capillary at a rate of 4 μl/sec to further control for radiation damage. Measurements were performed on a dilution series of PlyC alone from 3.3 to 0.14 mg/ml in a buffer consisting of 1 x phosphate-buffered saline with 10% (v/v) glycerol. Some concentration dependent oligomerisation was observed at protein concentrations above 1 mg/ml as evidenced by increases in Rg and disproportionate increases in I(0) (data not shown). The SAXS data used in this study were from protein at 0.9 mg/ml. Dilution of the protein below these concentrations did not result in changes to the shape of the scattering curve and calculated molecular weights at these concentrations were consistent with monomeric protein. The molecular weights of the scattering species were estimated from the total forward scatter of the SAXS measurements that were normalised by comparison to water scatter and with reference to the measured protein concentrations. Partial specific volume and scattering length density were calculated using the program MULCh [17]. The monomeric state of the protein was inferred by comparison of the theoretical molecular weight of the protein sequence with the calculated molecular weight from the SAXS experiment. A 1.6 m camera was used with an X-ray energy of 11 KeV giving a Q range from 0.01 to 0.5 Å-1. Data was collected on a Pilatus 1M detector (Dektris) and averaging of images, subtraction of blanks and radial integration was performed using the beamline control software ScatterBrain (Australian Synchrotron). Measurements were made at 25°C. Calculation of scattering intensities from molecular models was done using CRYSOL [18–19].

### All-atom Molecular Dynamic Simulations

MD simulations of three systems (PlyC complex, CHAP domain, and GyH domain) were performed using NAMD v2.8 [20] in conjunction with the CHARM22 forcefield with CMAP correction [21]. Chains 1 and A-H from the 3.30 Å structure of the PlyC complex (PDB accession ID 4F88) were used as a template for all simulations and missing residues were modelled using multiple rounds of Modeller 9v10.

PlyC complex: The protein complex was solvated with 35055 TIP3P water molecules in a rhombododecahedral box with a minimum padding of 10 Å and neutralised by the addition of 10 Na^+^ ions. The final system contained 121038 atoms.

GyH domain: The PlyCA GyH domain (Chain 1; Residues 1–209) was solvated with 12617 TIP3P water molecules in a cubic box with a padding of 10 Å and neutralised by the addition of 11 Na^+^ ions. The final system contained 40986 atoms.

CHAP domain: The PlyCA CHAP domain (Chain 1; Residues 302–465) was solvated with 8033 TIP3P water molecules in a cubic box with a padding of 10 Å and then neutralised by the addition of 4 Na^+^ ions. The final system contained 26529 atoms.

All MD simulations were performed in NPT conditions. A Langevin thermostat with a damping coefficient of 0.5 ps^-1^ was used to maintain the system temperature (300 K). The pressure was maintained at 1 atm using a Langevin piston barostat. Periodic boundary conditions were applied. The particle mesh Ewald algorithm was used to compute long-range electrostatic interactions. Nonbonded interactions were truncated smoothly between 10 Å and 12 Å. All covalent hydrogen bonds were constrained by the SHAKE algorithm allowing an integration time step of 2 fs.

In all simulations the initial system was subjected to conjugate gradients minimisation (5,000 steps) in the presence of harmonic constraints on the protein heavy atoms. This was followed by an equilibration step where the constraints were gradually removed for 5 ns. In the case of the PlyC complex care was taken to prolong this equilibration step by 50 ns to allow the modelled missing residues of the CHAP linker region to better equilibrate (Chain 1; Residues 288–310).

Three independent simulations of 1,100 ns were generated for the PlyC complex, two simulations of 1,000 ns for the GyH domain, and two simulations of 650 ns for the CHAP domain.

### MD analyses

Trajectories were visualised using VMD and analysed using MDTraj. Analyses were performed on the productive plateau stage of the simulations (i.e. after equilibration of systems or the first 100 ns of the simulation). Root Mean Square Deviation (RMSD) analysis was performed by calculating the positional deviations of backbone heavy atoms with respect to their initial structure every 2 ns (after performing a least-squares fit to the initial structure). Root Mean Square Fluctuation (RMSF) analysis was performed by calculating the fluctuations of C-alpha atoms with respect to their average positions every 2 ns (after performing a least-squares fit to their initial positions). Analysis of the hydrogen bonds employed a donor-acceptor cutoff distance of 3.5 Å and acceptor-donor-hydrogen cutoff angle of 30°.

For principal component analysis: the positional deviation of all atoms with respect to their average structure was calculated every 2 ns (after performing a least-squares fit to their initial positions). Scikit-learn’s [22] singular value decomposition procedure was then used to determine the eigenvectors and eigenvalues corresponding to the first 10 principal components of motion.

#### Normal modes calculation

Normal modes were calculated in vacuum using the CHARMM22 forcefield [23] with a distance dependent dielectric constant (*ε = 2r*_*i*,*j*_), to treat electrostatic interactions. Prior to NM calculations, the PlyC crystal structure was energy minimized using the steepest descent (SD) and conjugate-gradient (CG) methods followed by the Adopted Basis Newton Raphson (ABNR) algorithm. Harmonic restraints were applied during the SD steps and were progressively decreased from 250 to 0 kcal mol^-1^Å^-2^. Subsequently, the system was further energy minimized with 1000 CG steps and then applied the ABNR algorithm without positional restraints using a convergence criterion of 10^−5^ kcal mol^-1^Å^-1^ RMS energy gradient. The normal modes and the atomic fluctuations were computed with the VIBRAN module of CHARMM.

The degree of collectivity of a protein motion can be understood as a fraction of atoms participating to a given displacement [24]. For a mode vector of length 3*N* with elements *α*_*i*_, the degree of collectivity κ is defined as:

$$\kappa =\frac{1}{N}\exp\left(-\sum_{i=1}^{3N}\alpha_{i}^{2}\log\alpha_{i}^{2}\right)$$

If the conformational change involves only a few atoms, κ is minimal. On the other hand, if κ = 1 the given motion is maximally collective.

#### Free energy landscape (FEL) analysis

The free energy difference (ΔG_α_) of a particular state α with respect to the most populated one, was calculated according to the probability of finding these states as given by:

$$G_{\alpha }=-k_{B}T\;\ln\;\left[\frac{P(q_{\alpha })}{P_{max}(q)}\right]$$

where *k*_*B*_ is the Boltzmann constant, *T* the temperature of the simulations, *P*(*q*_*α*_) an estimate of the probability density function obtained from bi-dimensional histograms of the RMSD and radius of gyration values (here assigned as the reaction coordinates *q*_*i*_ and *q*_*j*_ *)*from each snapshot; *P*_max_(*q*) is the probability of the most visited state. Prior to the calculations, the three independent PlyC complex trajectories were concatenated and the FEL was obtained from the joint probability distributions *P(q*_*i*_, *q*_*j*_ *)*.

## Results and Discussion

### The structure of PlyC in the crystal and in solution are different

The PlyC crystal structure provided pivotal information with regard to the domain architecture and arrangement but failed to provide mechanistic information due to the occlusion of both active sites. These data suggested we had captured an auto-inhibited conformation of the enzyme. This was a surprising result given the recombinant PlyC protein in solution is remarkably stable and shows potent activity for extended periods of time (activity is retained for months upon cold storage). Control of lysin activity is, however, a biological necessity during the phage life-cycle to ensure that premature or uncontrolled host cell lysis does not occur [25]. Lysins accumulate in the cytosol of the infected bacteria in a fully folded state during the vegetative cycle[25]. At a specified trigger (post-assembly of progeny virion), the membrane is permeabilized to the lysin to allow destruction of the peptidoglycan layer. It is feasible, therefore, that within the intracellular environment, the lysin, waiting for access to its substrate, may adopt or sample a low-energy conformational state. It is possible that this state is what we have captured in our crystal structure.

**Fig 2:**
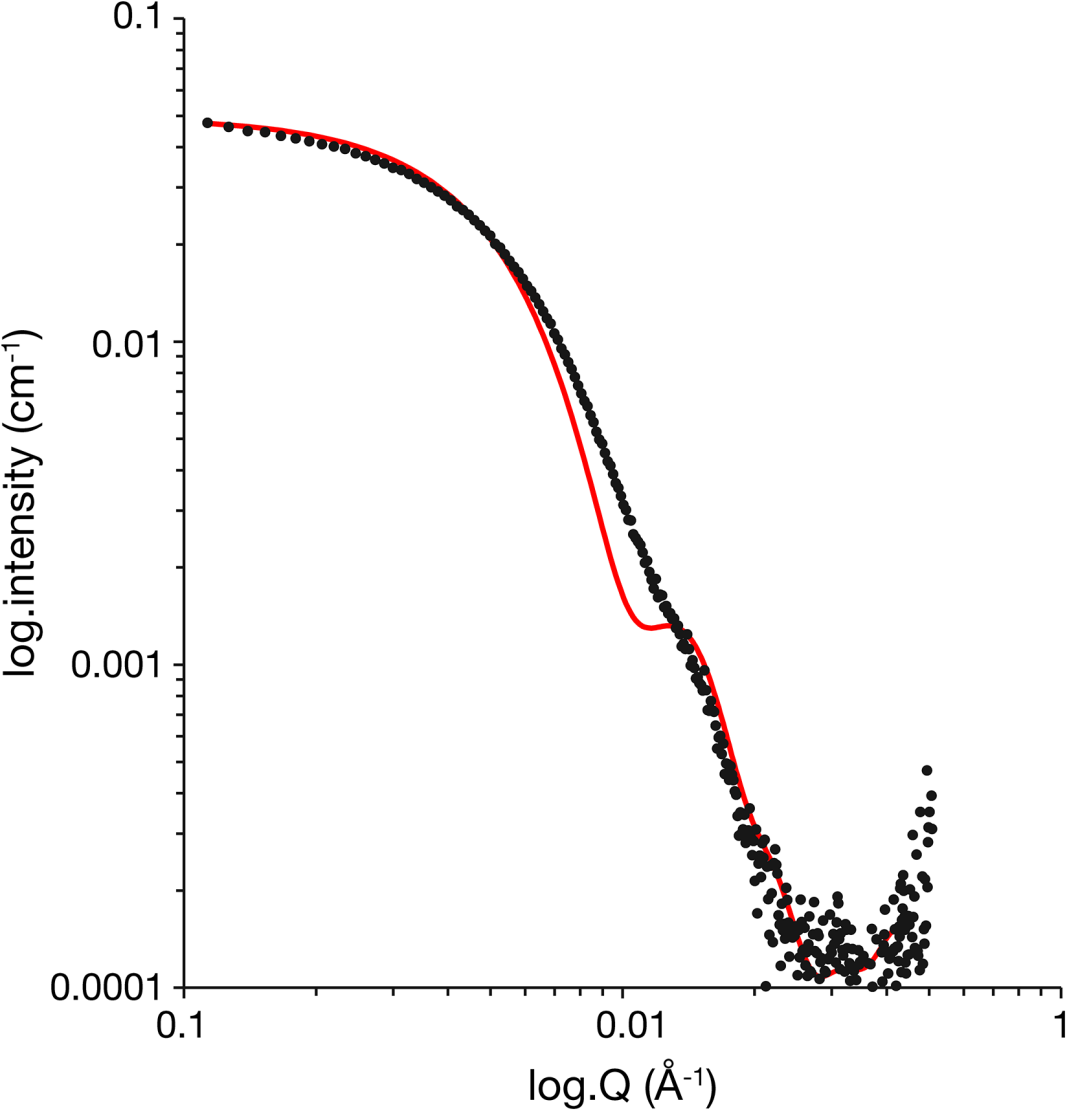
SAXS analysis of PlyC. **(A)** Comparison of the PlyC SAXS profile (black) and the theoretical scattering curve (red) calculated from the X-ray crystal structure of PlyC. The theoretical profile was calculated and fitted to the experimental curve using CRYSOL (Svergun et al. 1995).

However, we were interested in the mechanism of PlyC action and we hypothesized that our crystal structure did not represent the shape or arrangement of the active protein complex [10]. To answer this question, we investigated the solution structure of active PlyC using small angle X-ray scattering (SAXS) analysis. Comparison of the PlyC SAXS profile with the theoretical scattering profile calculated from the crystal structure indicates that the shape of PlyC in solution and the crystal structure are indeed different (the *χ2* for the fit = 4.1; Fig 2). The radius of gyration and molecular mass derived directly from the SAXS data are in good agreement with the crystal structure (Table 1) indicating that this poor fit does not arise as a result of multimerization. This assertion is strengthened by the observation that there is no concentration dependence of Rg or MW at concentration below 1.8 mg/ml (Table 1). The major point of difference is a peak at Q ~ 0.13 Å that is weak and diffuse in the experimental data but rather pronounced in the data calculated from the crystal structure. This peak is likely to correspond to vectors between the various sub-domains of the PlyC enzyme that, in the crystal structure, are separated by discrete distances with little intervening density.

**Table 1:** Parameters used for SAXS analysis. The table shows the parameters used for collection of the SAXS data, the structural parameters obtained from SAXS analysis and the software employed to perform the analysis

**Data collection parameters**	
Instrument	Australian Synchrotron SAXS/WAXS beamline
Beam geometry Wavelength (Å)	120 micron point source, 1.0333 Å
q-range (Å^-1^)	0.034 to 0.45 Å^-1^
Exposure time (min) Concentration range (mg ml 1)	10x 1 sec exposures with sample flow at 4
Temperature (K)	ml/sec, 0.9 mg/ml, 25°C
**Structural parameters**	
I(0) (cm^-1^) [from P(r)]	4.67E-02
Rg (Å) [from P(r)]	31.69
I(0) (cm^-1^) (from Guinier)	4.70E-02
Dmax (Å)	110
Rg (Å) (from Guinier)	31.75
Calculated Rg from structure (Å)	31.8
Porod volume estimate (Å^-3^)	109239
Dry volume calculated from sequence (Å^-3^)	88,025
**Molecular-mass determination**	
Partial specific volume (cm^3^ g^-1^)	0.737
Contrast (Δp x 10^30^ cm^-2^)	2.86E+10
Molecular mass M_r_ [from I(0)]	87.7
Calculated monomeric M_r_ from sequence (KDa)	89.4
**Software employed**	
Primary data reduction	ScatterBrain (Australian Synchrotron)
Data processing	PRIMUS/GNOM
Ab initio analysis	n/a
Validation and averaging	n/a
Rigid-body modelling	n/a
Computation of model intensities	CRYSOL
Three-dimensional graphics representation	n/a

One possible explanation for the difference between the X-ray crystal structure and the SAXS data could be that the conformation of PlyC observed in the crystal structure was influenced by crystal lattice contacts. To investigate the effect of crystal packing on the structure of PlyC, we analysed the crystal contacts in the structure (PDB ID: 4F88). We found 12 hydrogen bonds between the two PlyC structures present in the asymmetric unit and their symmetry partners (S1 Fig). Three of these hydrogen bonds were between the CHAP domain in PlyCA and the PlyCB ring in a symmetry partner. These lattice contacts, together with the cryo temperatures used, may affect the position of the CHAP domain and hence also the overall conformation of PlyC, effectively “locking down” the CHAP domain, offering one explanation for why PlyC appears more compact in the crystal structure than in solution.

### PlyC dynamics involves concerted domain motions

**Fig 3:**
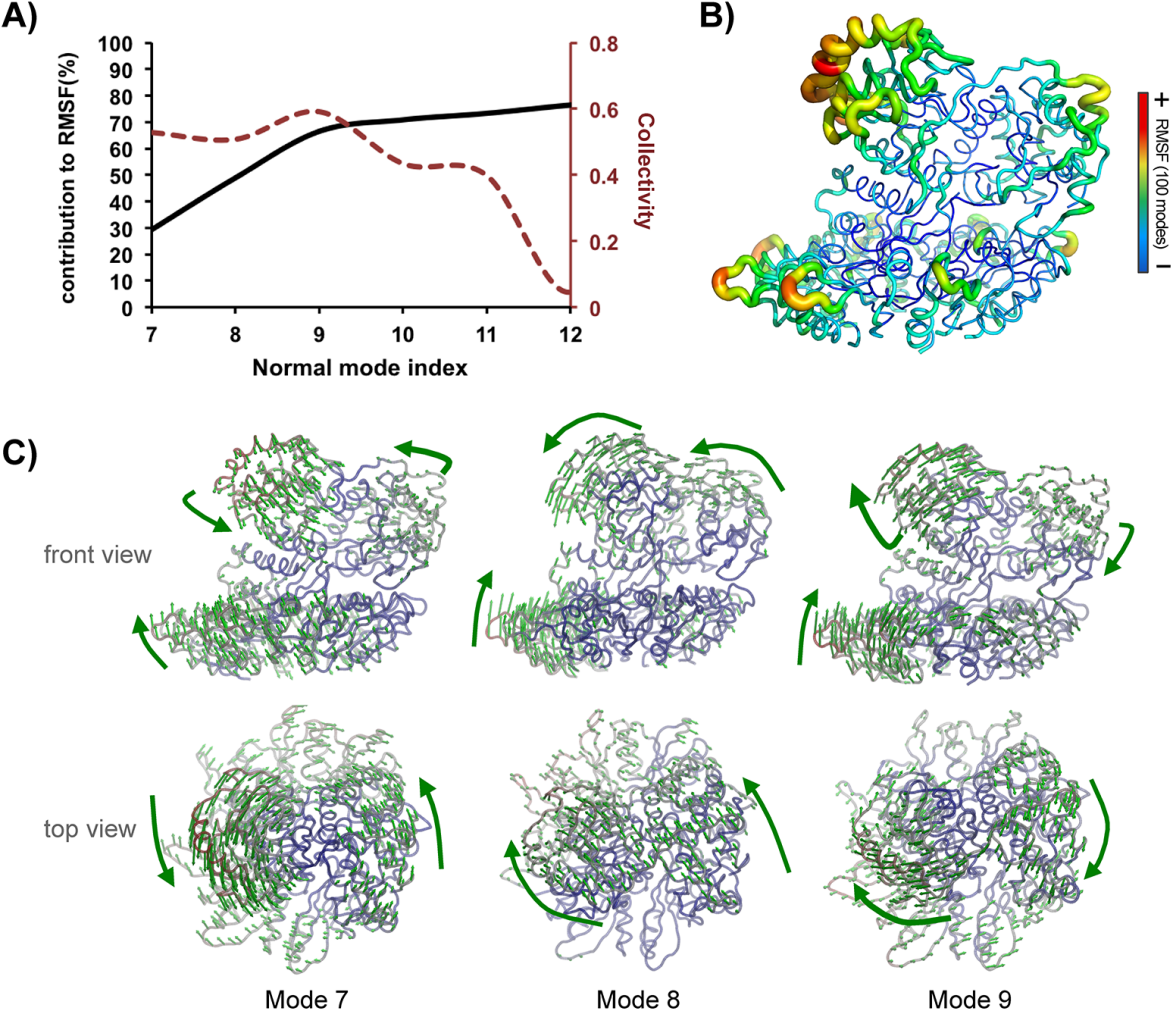
Normal mode analysis of the PlyC complex. **(A)** Cumulative contribution by the first five low frequency modes to overall fluctuations (left axis); degree of collectivity by mode (right axis); **(B)** total flexibility contributed by the first 100 normal modes embedded within PlyC structure. The width of the tubes and colors are proportional to the magnitude of the RMS fluctuations; **(C)** The directions of motions described by the first three normal modes (7 to 9) are represented by arrows (atoms moving less than 1Å were omitted to improve clarity).

We were next interested in exploring the intrinsic flexibility encoded in the PlyC holoenzyme. To this end, we performed a normal mode (NM) analysis, which allowed us to inspect motions related to the low frequency end of the dynamic spectrum. Fig 3A clearly shows that overall PlyC dynamics are governed by the first three internal modes (7 to 9, since the six first modes correspond to overall translation and rotation motions). Together, the cumulative contribution of these modes to the overall fluctuations is approximately 70%. Inspecting the accumulated contribution of the first 100 modes to the RMS fluctuations, the CHAP domain is the most flexible region of the complex, followed by the GlyH domain and loops within the PlyCB ring (Fig 3B and S1 Movie).

Given the known importance of collective motions in protein functions [24] we computed the degree of collectivity of each mode (defined in Methods). We identified the first three modes as the most collective ones, presenting values markedly higher than the subsequent ones (10–12). We inspected the directions of motions described by each of these three modes (Fig 3C). Clearly, there is significant conformational plasticity of the CHAP and GlyH domains, which appears to move in a concerted fashion. Furthermore, the PlyCB ring moves synergistically with the upper PlyCA domains, raising the possibility that such complex dynamics may be transmitted throughout the complex structure (S1 Movie).

### MD simulations reveal a highly mobile multi-domain architecture

Since NM analysis allowed the identification of the intrinsic PlyC motions, we were interested in further exploring the conformations adopted in solution. To this end, we carried out all-atom MD simulations, in triplicate, of the entire PlyC complex as well as the catalytic GyH and CHAP domains in isolation, in order to provide insights into the conformational space explored by these proteins.

**Fig 4:**
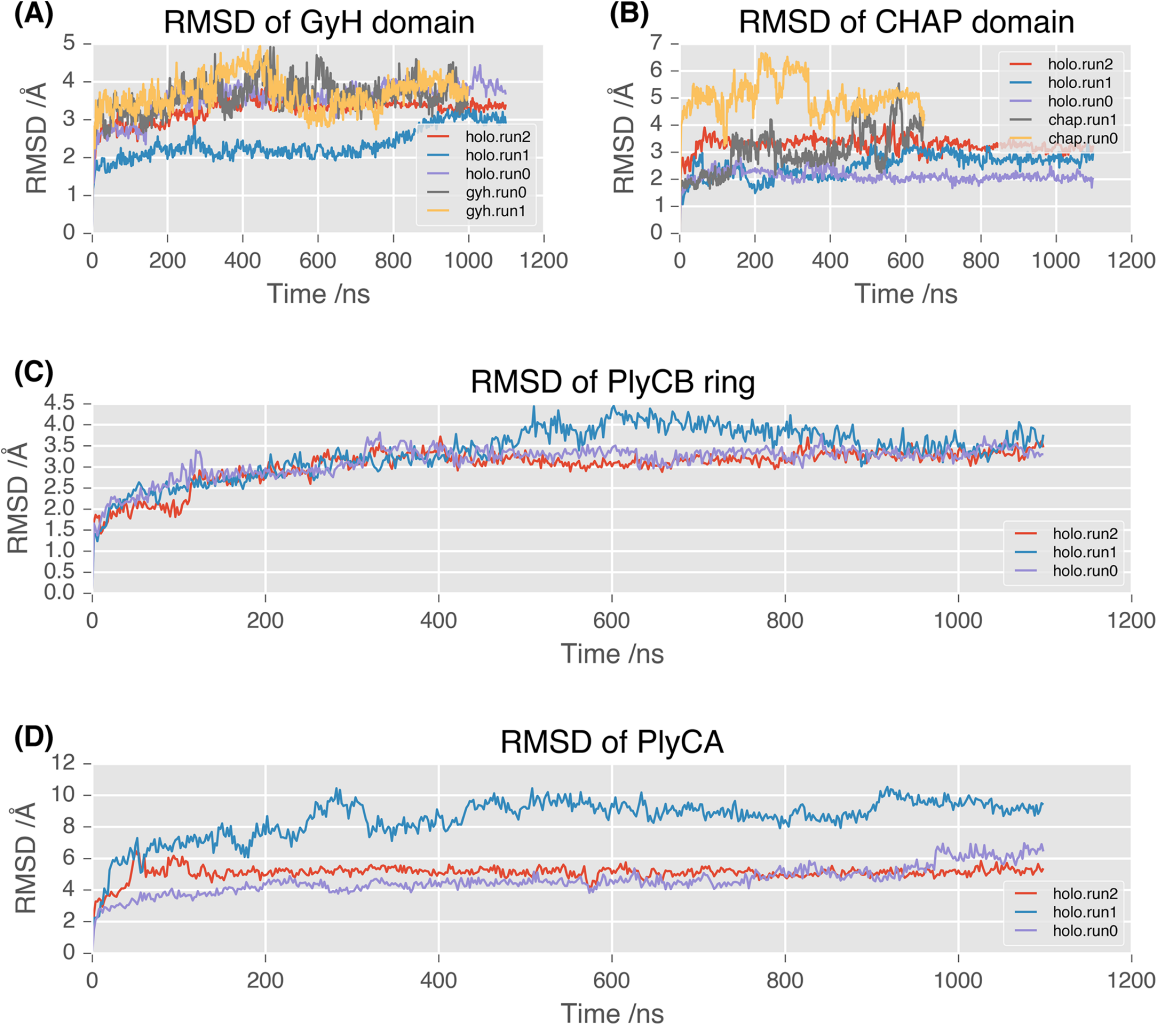
Dynamic behaviour of PlyCA and PlyCB subunits. RMSD of backbone atoms in MD simulation trajectories against initial coordinates/position, calculated for **(A)** GyH domain alone; **(B)** CHAP domain alone; **(C)** PlyCB ring (8 chains); **(D)** PlyCA chain alone.

Inspection of the time evolution of the root mean-square deviations (RMSDs) of the PlyC complex suggests moderate flexibility within the GyH (Fig 4A) and CHAP (Fig 4B) domains, both reaching equilibrium in less than 50 ns. Average values of backbone RMSD for the productive stage (following the initial 50 ns) of each simulation are 3.09 ± 0.62 Å (GyH, 836 atoms) and 2.66 ± 0.56 Å (CHAP, 656 atoms). The PlyCB octameric ring is notably rigid, reaching equilibrium in 300 ns, with an RMSD for the production stage of 3.25 ± 0.41 Å across all 8 chains (2272 atoms, Fig 4C).

While the conformation of the polypeptide chain within individual PlyCA domains remained relatively invariant during simulation, the relative orientation of domains in the PlyCA complex did not. Whilst two of the simulation runs resulted in an RMSD over the entire PlyCA chain (1860 atoms) of 4.71 ± 0.75 Å and 5.16 ± 0.28 Å, the third run resulted in a much greater RMSD of 8.71 ± 0.97 Å, sampling a much larger conformational space (Fig 4D). Given the size of the system it is reasonable to expect that longer timescale simulations would be necessary to observe similarly large structural changes in the remaining replicates.

**Fig 5:**
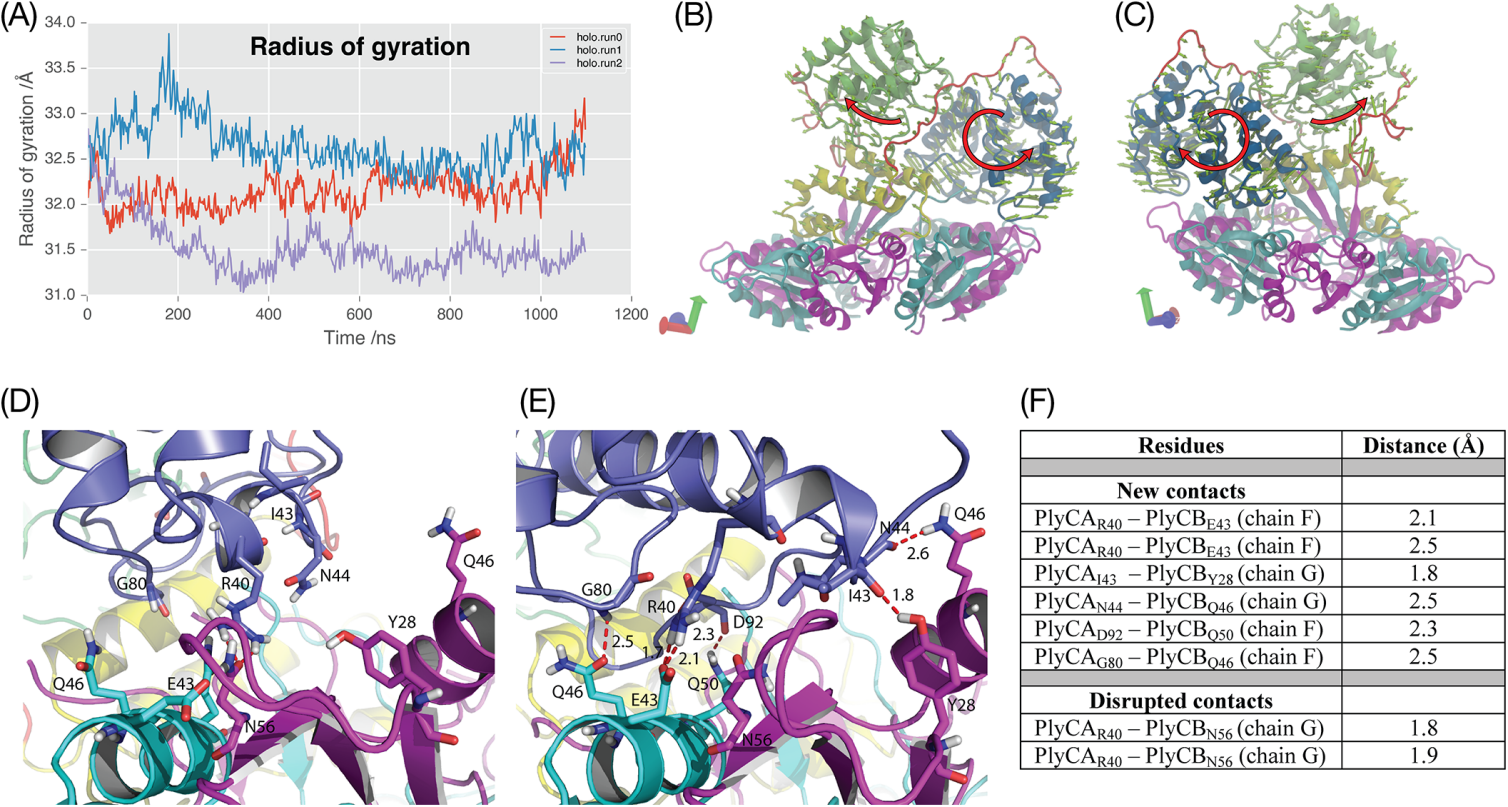
PC analysis of the MD trajectories. **(A)** Radius of gyration for the PlyC complex throughout simulation; (**B, C**) A single PC describes rotation of both GyH and CHAP domains; PC1 of trajectory run0. Standard view **(B)** and rotated 180° as indicated by arrow **(C)**; **(D, E)** View of the interface between the GyH domain and the PlyCB ring in the starting conformation (**D**) and (**E**) upon a maximum displacement along PC1. The hydrogen bonds are indicated by red dashed lines and the distance of the bond is given in Å. **(F)** Contacts that are disrupted and formed as a result of the first PC motion.

We next used principal component (PC) analysis to identify the most statistically relevant motions occurring during the simulations, which are not easily detected from a simple inspection of the trajectories. Eigenvectors and associated eigenvalues were found for the matrix of atomic positional deviations using singular value decomposition. The first five principal components (PCs) accounted for 70, 80 and 65 percent of the total motion in the trajectories (S1 Table). The first two major motions (PCs 1 and 2) in all trajectories described rigid-body motions of the catalytic domains, relative to the PlyCB ring. In these motions, the catalytic domains rotated between 15 and 45 degrees, but only experienced a maximum translational shift of 1.12 Å. Since both domains are approximately spherical in nature, there were no significant changes to the radius of gyration throughout the trajectory (Fig 5A, S2 Movie), which is consistent with the small difference in radius of gyration obtained from SAXS and the X-ray crystal structure (Table 1). The first two principal components describe the outward (i.e. away from the molecule’s centre of mass) rotation of both the GyH domain and the CHAP domain from their initial positions (Fig 5B, S2 Movie). As the GyH domain rotates, the lower side (closest to the PlyCB ring) rolls outwards and upwards, while the upper side moves downwards, towards the docking domain (Fig 5C, S2 Movie). These motions result in the loss of two hydrogen-bond contacts between the GyH domain and the PlyCB ring, and formation of several new hydrogen-bonded interactions between the GyH domain and the PlyCB ring (Fig 5D, 5E and 5F). Given previous point mutations have ruled out a functional role for PlyCB_Q46_ and PlyCB_Q50_[10], we hypothesize that this flux in hydrogen bonds between the two proteins reflects an opportunistic network that occurs as the catalytic domain rotates.

The CHAP domain rotation is accompanied by a large translocation of the CHAP linker region (residues 289–294), moving 11 Å away from the PlyCB ring (Fig 5C, S3 Movie). Movement of the CHAP linker is consistent with its demonstrated lability in solution, susceptibility to proteolytic digestion, and the lack of electron density in the crystal structure[10], indicating that it is freely accessible away from the body of the molecule.

Intriguingly, the catalytic domain rotations result in an overall decrease in the surface accessible solvent area (SASA) for the entire complex (ΔSASA ≈ -1500 Å^2^) in all replicates (S2 Fig). This is consistent with the domain reorganisation changing the shape of the molecule, consistent with our SAXS data.

### Dynamics of the PlyCB ring suggests information transfer to the catalytic domains

**Fig 6:**
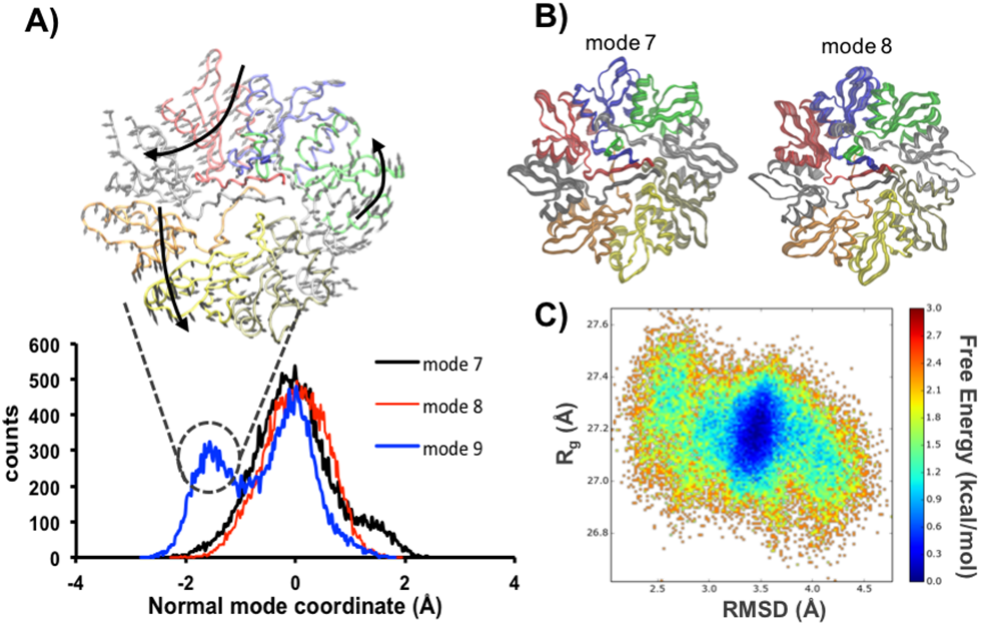
PlyCB dynamics in solution. **(A)** Projections of each conformation sampled during MD in explicit solvent onto NM vectors. The three MD trajectories were concatenated into a single one prior to the analysis. The structural transition associated to the population at -2 Å MRMS is depicted in the upper part of the graph. **(B)** Structural variation of the PlyCB ring along modes 7 and 8 shows asymmetric flexibility along each subunit. **(C)** Free energy landscape along the RMSD and Rg values computed over the concatenated trajectory.

Regarding the inherent dynamic nature of proteins, the evaluation of the conformational equilibrium in solution is more informative than classical analysis using a static perspective [26]. In line with this view, we calculated the distributions of the NM coordinates of each conformation sampled during our MD simulations (Fig 3). Based on the inspection of the populations obtained, we can estimate the extents of sampling by mode in explicit solvent, whose effects are not accounted on the NM calculations (Fig 6A). Here, only the Cα atoms of the PlyCB rings were considered in the calculation of the projections. The distributions obtained for modes 7 and 8 revealed in both cases a narrow population of conformations centred on the reference structure, which is indicative of limited sampling along these directions (Fig 6B). However, for mode 9 we observed two well-defined populations, one similar to those observed for the other modes and another 2 Å apart (on the MRMS scale) from the reference. The atomic motions related to such transition are depicted in the upper part of the plot (Fig 6A).

We also computed a free energy landscape using as reaction coordinates the RMSD and the radius of gyration (Fig 6C). Although we noticed a single low energy population, the large conformational space sampled do not present high energy barriers separating distant states, which indicates a substantial ability of PlyCB to undergo large conformational transitions as such described by NM analysis. Altogether, these findings show that the PlyCB ring may be able to (1) better adapt to irregular surfaces presented on the cell wall, and (2) acts not only as a docking motif to the cell wall, but also as a transmitter of dynamic information to the catalytic domains.

### Dynamics of catalytic domains suggests that synergy and potent activity are dependent on complete PlyC architecture

We compared the mobility of the GyH and CHAP catalytic domains in isolation with the behavior observed when they are attached to the complete protein scaffold. The CHAP domain is less mobile in the holoenzyme simulation than in isolation, rapidly stabilizing with an RMSD of 2.66 ± 0.56 Å in the holoenzyme, compared to an average RMSD of 4.12 ± 1.20 Å after 550 ns in isolation (Fig 4B). However, the overall mobility of the GyH domain was similar in both the holoenzyme and isolated domain simulations, stabilizing after 50 ns to an RMSD of 3.69 ± 0.41 Å from the initial structure in all simulations (Fig 4A).

**Fig 7:**
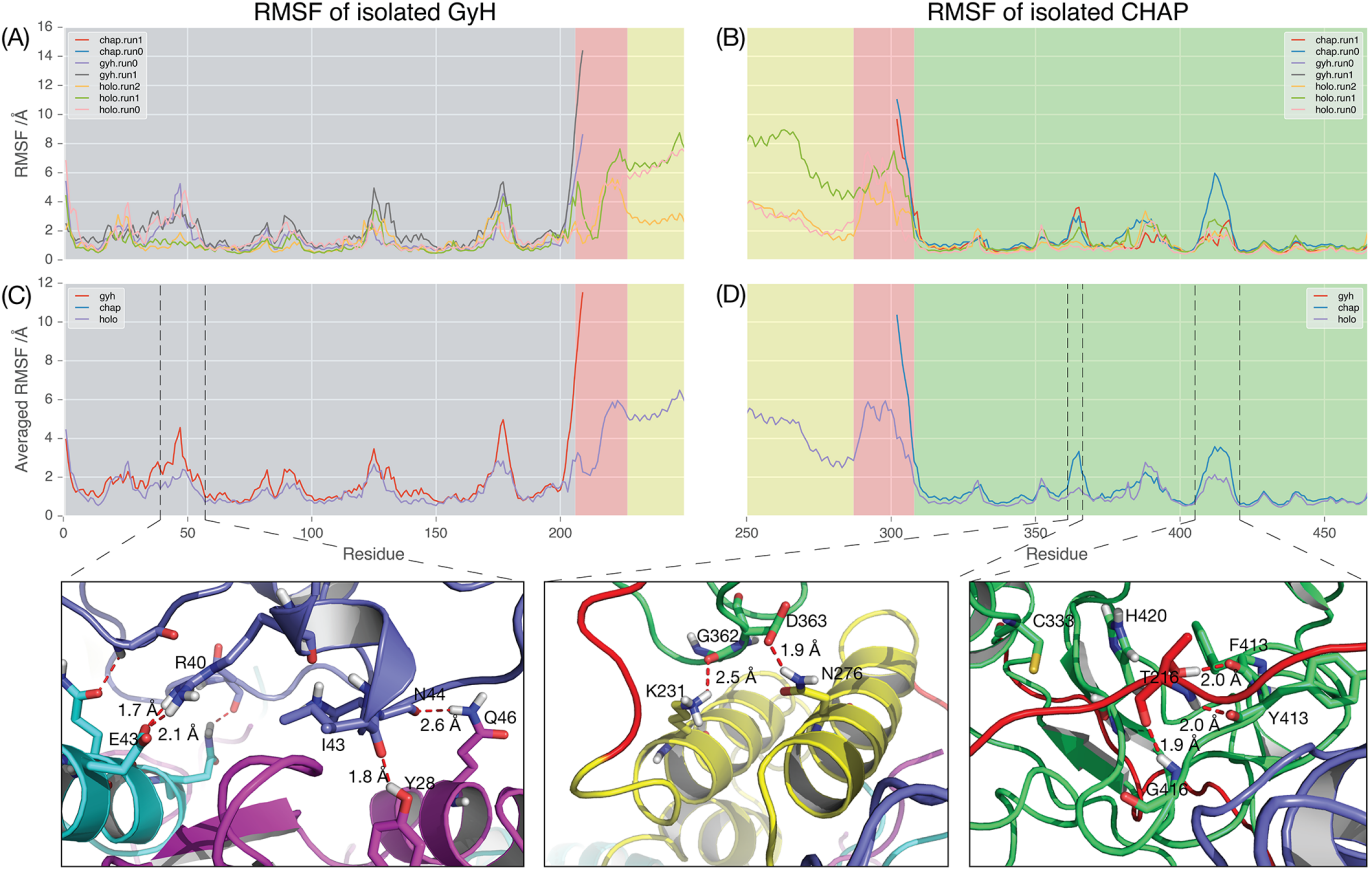
Interdomain interactions reduce the dynamics of the PlyC holoenzyme. Root mean square fluctuation (RMSF) of Cα for each residue in all simulations aligned to the GyH domain (**A**) or the CHAP domain (**B**); **(C, D)** Average RMSF of Cα for each residue over all simulations of the holoenzyme (purple, n = 3), the GyH domain (red, n = 2), and the CHAP domain (blue, n = 2) aligned to the GyH domain (**C**) or the CHAP domain (**D**). Specific hydrogen bonding interactions observed in the holoenzyme that are responsible for reduction in dynamics are shown below (taken from simulation snapshots).

We further investigated how the internal dynamics within domains is altered by interdomain interactions in the holoenzyme by comparing root mean-square fluctations (RMSFs) across the protein backbone during simulations of the individual domains with simulations of the entire holoenzyme (Fig 7). During the simulation of the PlyC holoenzyme, we observed that protein-protein interactions restrict the motion of a helical region (residues 40–55) in the GyH domain. The RMSF of this helical region is reduced from 4.6 Å in simulations of GyH alone to only 2.4 Å in simulations of the holoenzyme (Fig 7C). This is a likely result of the outward rotation of the GyH domain and change in its interactions with the PlyCB ring (Fig 5B).

Two distinct regions of the CHAP domain have motions also dampened by protein-protein interactions within the holoenzyme. Notably, these regions are involved in formation of the active site pocket (residues PlyCA_405-420_ and PlyCA_360–365_; Fig 7D). In isolation, residues PlyCA_405-420_ have a maximum RMSF of 3.6 Å during simulation, but only 2.1 Å in the holoenzyme. The motion of this region in the PlyC holoenzyme appears restricted by inter-domain contacts between PlyCA_F413, Y414, G416_ in the CHAP domain and PlyCA_T216_ in the GyH linker (Fig 7D). Interestingly, this residue range also includes PlyCA_T406,_ which was recently identified as a stabilising position for the protein scaffold. Alteration of the Thr to Arg resulted in a 16-fold increase in kinetic stability[27]. Finally, in isolation, residues PlyCA_360–365_ have a maximum RMSF of 3.3 Å, but only 1.5 Å in holoenzyme, again due to ionic intra-domain interactions, this time between CHAP domain residues PlyCA_G362_ (backbone) and PlyCA_D363_ with PlyCA_K231_ and PlyCA_N276_ from the docking domain, respectively.

#### MD simulations provide glimpses of a substrate-ready PlyC conformation

Synergy and/or cooperation between the catalytic domains of PlyCA are thought to significantly contribute to the enzyme’s enhanced lytic activity [10]. Our findings indicate that dynamics may play a significant role in communication between the domains. Therefore we next investigated how the dynamics affects the active sites of the catalytic domains, and specifically how the change in solvent accessibility of catalytic residues during simulation may provide insights into their readiness to cleave substrate.

**Fig 8:**
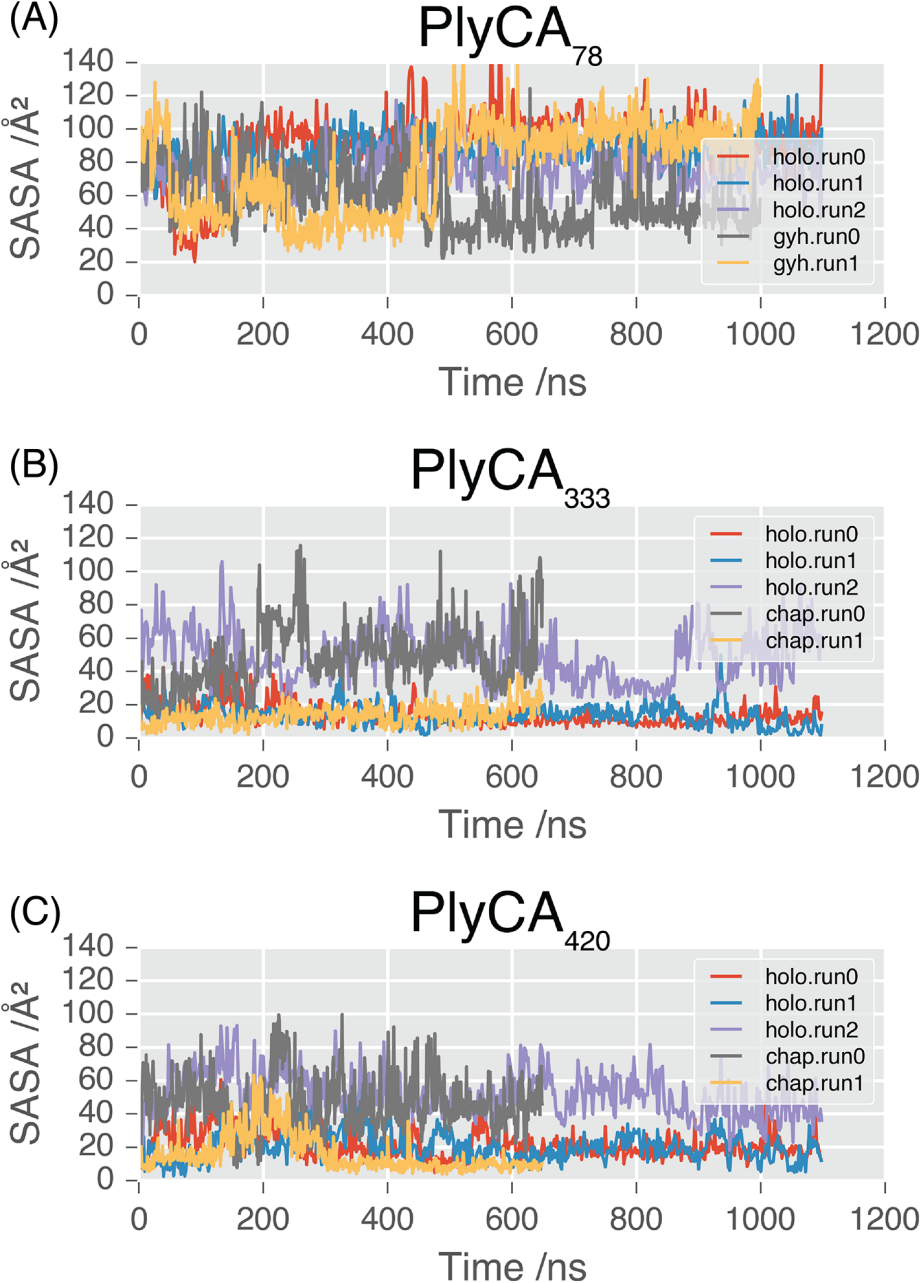
SASA of the catalytic residues in PlyC. Solvent accessible surface area associated with catalytic residues throughout simulation: (**A**) PlyCA_E78_, (**B**) PlyCA_C333_, (**C**) PlyCA_H420_.

The GyH domain is a member of class IV family 19 chitinases [10]. Alignment of the GyH domain identified that PlyCA_E78_ corresponds to a key catalytic position within the family 19 chitinases [10]. During the simulations of the GyH domain in isolation, PlyCA_E78_ fluctuates between exposed (SASA = 90 Å^2^), and partially exposed (SASA = 40 Å^2^) (Fig 8A). In run0, it is initially exposed, and then becomes buried at 450 ns. In run1, it is initially exposed, becomes buried at 70 ns, and then becomes partially accessible to solvent again at 500 ns. In contrast, whereas in the crystal structure of the PlyC holoenzyme PlyCA_E78_ is partially exposed (SASA = 58 Å^2^), in simulations of the PlyC holoenzyme it rapidly becomes permanently solvent accessible (SASA = 90 Å^2^; Fig 8A).

In the CHAP domain, the active site residues are PlyCA_C333_ and PlyCA_H420_[9]. In simulations of the CHAP domain alone, PlyCA_C333_ became solvent exposed (50 Å^2^) after 190 ns in one run, but remained buried in the second simulation (10 Å^2^). In simulations of the holoenzyme, PlyCA_C333_ was solvent exposed in one of three replicates (50 Å^2^), but remained buried in the other two (Fig 8B). The solvent accessibility of PlyCA_H420_ followed similar trends: in one out of the three simulations of the PlyC holoenzyme and one out of the two simulations of the CHAP domain, this residue was solvent-exposed (50 Å^2^), but remained buried (20 Å^2^) in the remainder of runs (Fig 8C).

We previously postulated that the helical docking domain of PlyCA plays an important role in stabilizing the interaction between the PlyCA and PlyCB subunits during engagement of the PlyCB ring with the cell wall [10]. It is notable that the helical docking domain remains relatively rigid during our MD simulations, displaying a peak backbone RMSF of 1.34 Å (S3 Fig). Our MD data therefore supports this hypothesis in that the PlyCB ring must remain relatively rigid to secure cell wall binding, yet in contrast the GyH and CHAP domains require mobility in order to achieve correct positioning for catalytic efficiency. The docking domain may therefore act as an adaptor between these static and dynamic elements. Indeed, the NM analysis suggests possible dynamic communication between the PlyCA and PlyCB subunits (Fig 3), that may play an important role in coupling cell-wall binding to peptidoglycan cleavage.

Taken together, the simulations provide intriguing glimpses into how domain dynamics may alter the active sites of the catalytic domains to allow binding to substrate. Peptidoglycan represents a significant challenge for efficient enzyme hydrolysis. Peptidoglycan is a hetero-polymer of linear glycan strands cross-linked by peptides in a net-like fashion. The peptidoglycan sacculus, however, is not the rigid exoskeleton once depicted in textbooks but a dynamic structure with considerable plasticity[28]. The capacity to (i) engage, (ii) hydrolyse, and (iii) continue hydrolysis in a processive manner demands a flexible and adaptable enzyme. The movement of the catalytic domains of PlyC as we have observed via MD shows that the enzyme may be sampling a substrate-ready conformation. A complete understanding of the conformational changes required for PlyC activity will require atomic knowledge of the peptidoglycan engagement by the catalytic domains as well as MD simulations on a much longer timescale, possibly milliseconds.

## Conclusions

In this study we have used SAXS and MD simulation to gain further insight into how the dynamic behavior of PlyC governs its extraordinary catalytic efficiency. SAXS reveals that the conformational ensemble of PlyC in solution is significantly different than in the crystal structure. Our NM and MD observations imply that the PlyC holoenzyme may use the inherent dynamics within the catalytic domains to drive synergistic and/or cooperative inter-domain communication that underpins its remarkable potency. Although our investigation reveals tantalizing glimpses into PlyC dynamics, important mechanistic questions remain. Notably, the question of substrate induced conformations and the role of substrate binding on the synergistic or cooperative action of the catalytic GyH and CHAP domains. Nonetheless, our study represents the first detailed investigation into the role of dynamics in PlyC function, providing experimentally-testable hypotheses as well as a framework for further exploration.

## Supporting Information

S1 Fig Crystal packing in the asymmetric unit of the PlyC crystal unit cell. 12 hydrogen bonds between the two PlyC structures present in the asymmetric unit and their symmetry partners are shown by red bonds.
(PNG)

S2 Fig SASA calculated from PlyC complex trajectories. (PNG)

S3 Fig The docking domain of the PlyCA chain is rigid. Root mean square fluctuation (RMSF) of Cα by residue in all simulations, after least-squares alignment to the docking domain (PlyCA, residues 228–286).
(PNG)

S1 Movie Motions described by low frequency normal modes. Representation of a 2Å MRMS (mass weighted root mean square) displacement along modes 7 to 9. The PlyC complex structure is colored by the magnitude of the fluctuations contributed by each mode, where blue tubes represent rigidity, white tubes represent moderate flexibility and red tubes highlight the most mobile regions.
(MP4)

S2 Movie Rigid-body rotations of the catalytic domains in all three MD simulation trajectories (MP4)

S3 Movie The movement of the CHAP domain. The movement is restricted to the CHAP linker region (residues 289–294), shows an 11 Å move away from the PlyCB ring.
(MP4)

S1 Table Percentage of variance captured in first five principal components. This table shows the explained variance ratio for each principal component in the trajectories.
(DOCX)
