# Supplementary figures and images for: Dynamic Motion and Communication in the Streptococcal C1 Phage Lysin, PlyC
Source: PLoS One. 2015 Oct 15;10(10):e0140219. doi: 10.1371/journal.pone.0140219 (PMC4607406; doi:10.1371/journal.pone.0140219)

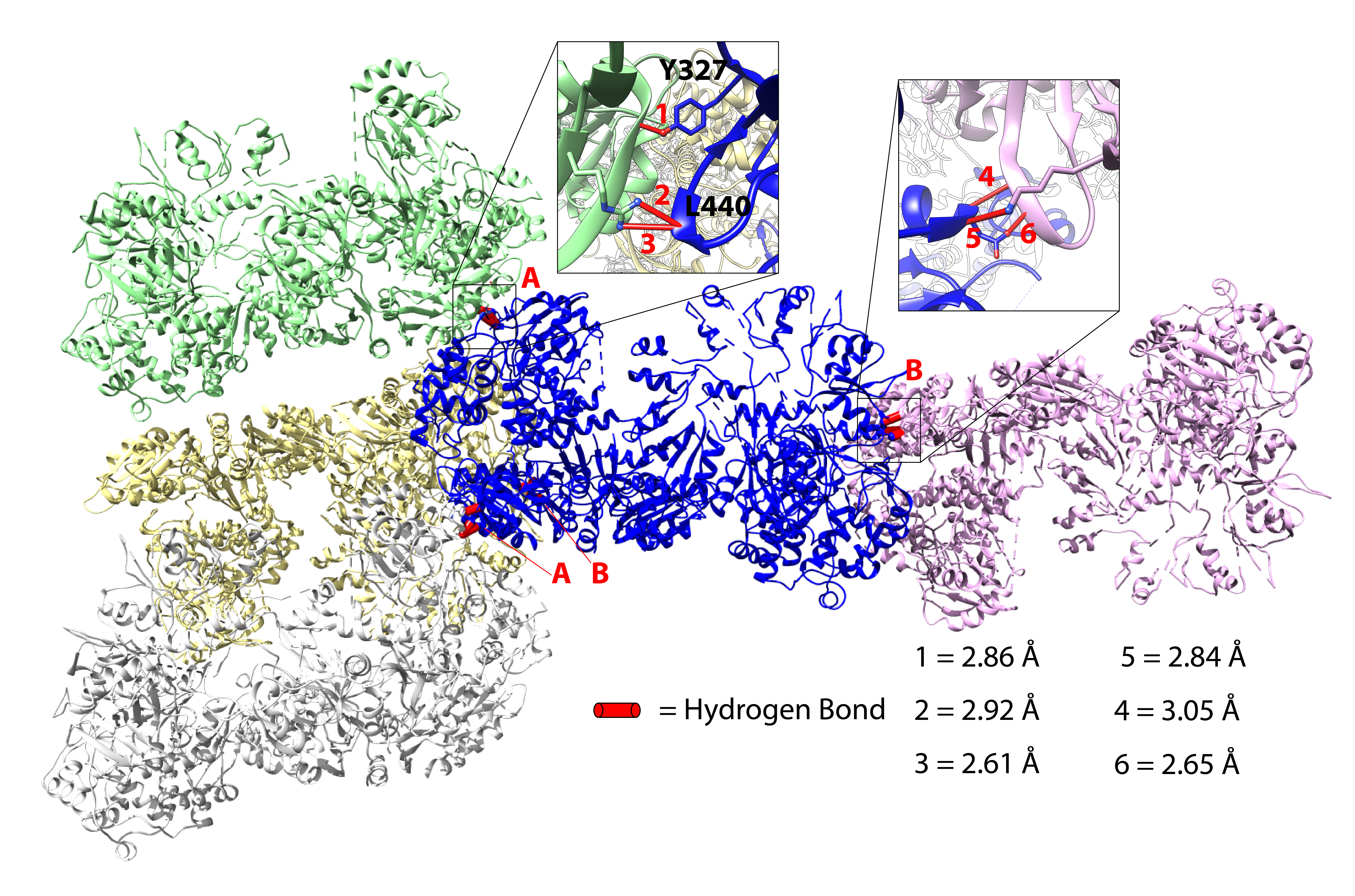

Supplement: S1 Fig — 12 hydrogen bonds between the two PlyC structures present in the asymmetric unit and their symmetry partners are shown by red bonds. (PNG) [file pone.0140219.s001.png]

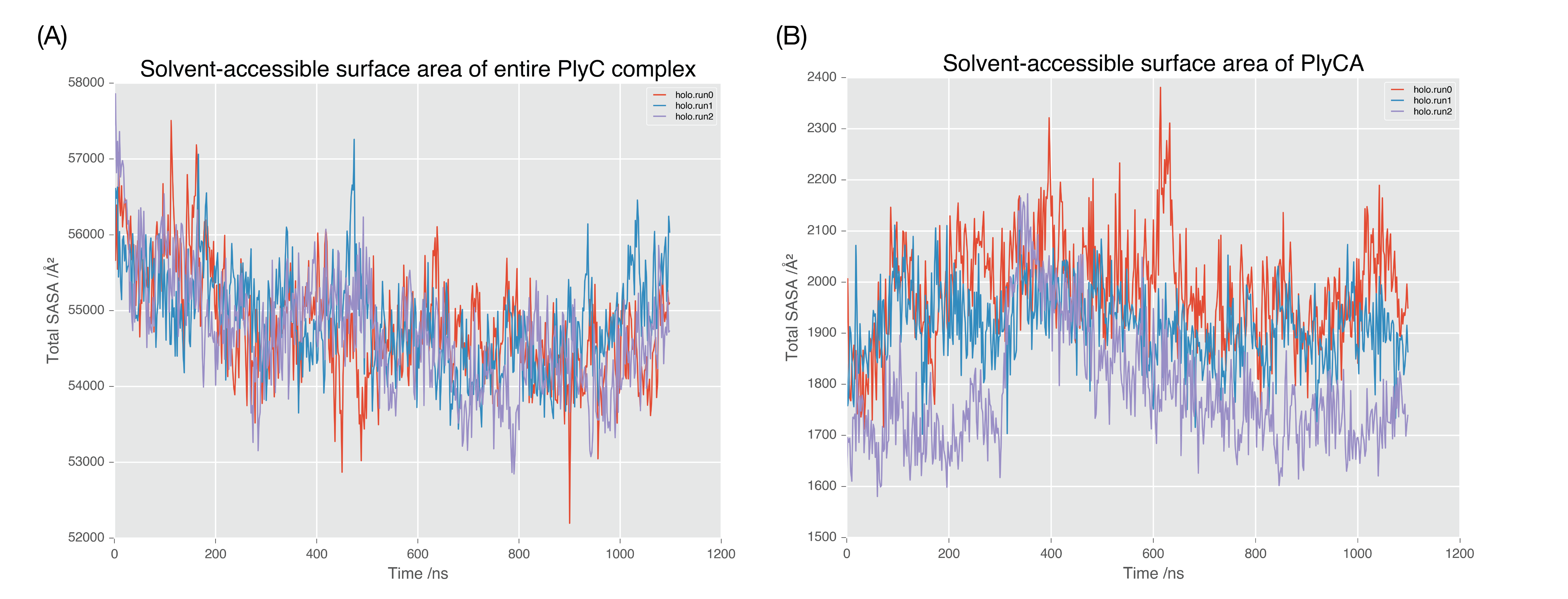

Supplement: S2 Fig — (PNG) [file pone.0140219.s002.png]

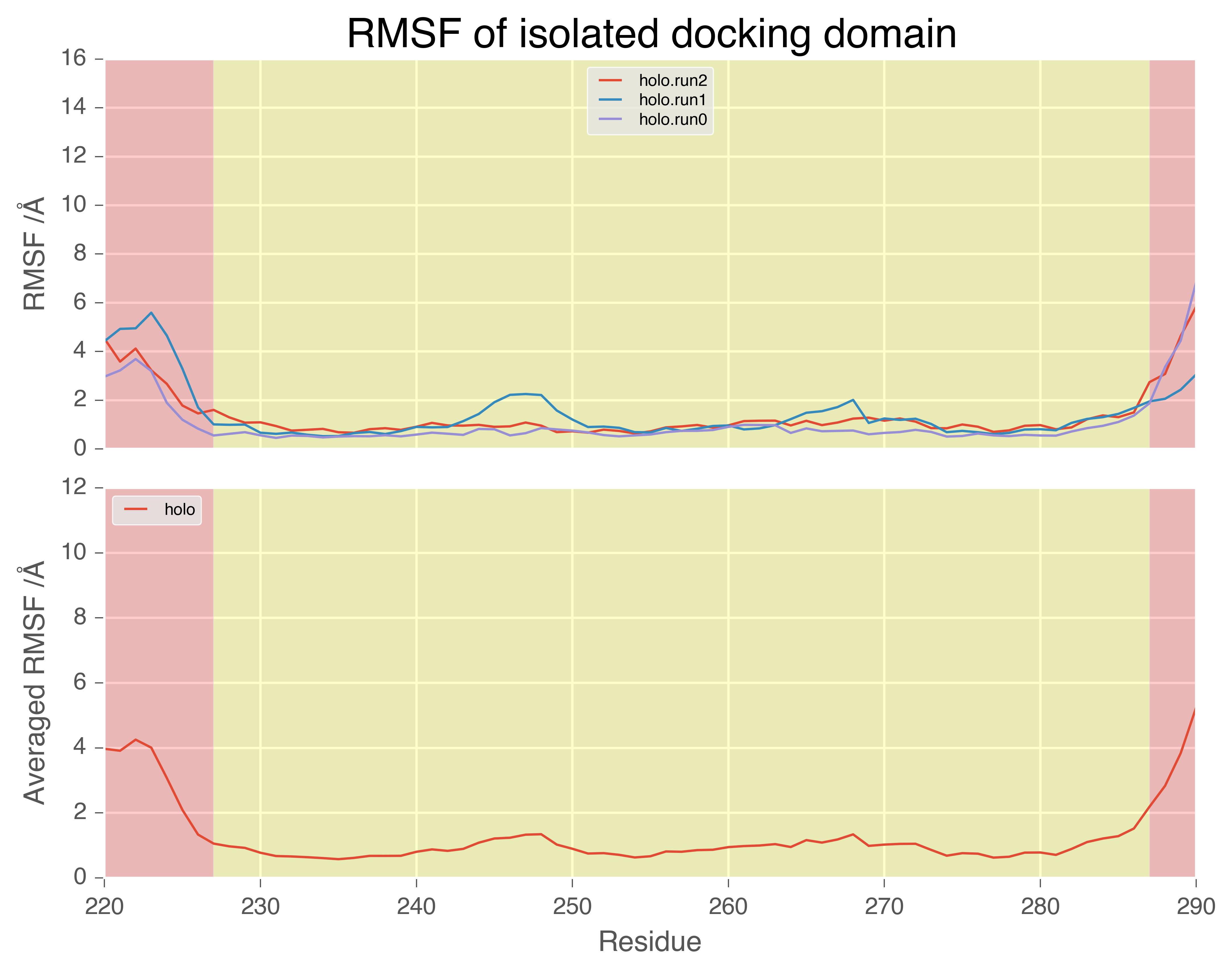

Supplement: S3 Fig — Root mean square fluctuation (RMSF) of Cα by residue in all simulations, after least-squares alignment to the docking domain (PlyCA, residues 228–286). (PNG) [file pone.0140219.s003.png]
