# Supplementary material for: Dynamic Motion and Communication in the Streptococcal C1 Phage Lysin, PlyC
Source: PLoS One. 2015 Oct 15;10(10):e0140219. doi: 10.1371/journal.pone.0140219 (PMC4607406; doi:10.1371/journal.pone.0140219)
Supplement: S1 Table — This table shows the explained variance ratio for each principal component in the trajectories. (DOCX) [file pone.0140219.s007.docx]

**Table S1. Percentage of variance captured in first five principal components.** This table shows the explained variance ratio for each principal component in the trajectories. Only the first five principal components are shown and therefore do not total 100 %.

| Principal component | 1 | 2 | 3 | 4 | 5 | Σ PC1—5 |
| --- | --- | --- | --- | --- | --- | --- |
| holo.run0 | 37.64 | 14.82 | 6.97 | 5.34 | 4.75 | 69.52 |
| holo.run1 | 49.74 | 14.70 | 8.44 | 4.34 | 2.85 | 80.08 |
| holo.run2 | 37.39 | 11.72 | 8.24 | 4.73 | 3.37 | 65.44 |
